# Advancement on Milk Fat Globule Membrane: Separation, Identification, and Functional Properties

**DOI:** 10.3389/fnut.2021.807284

**Published:** 2022-01-28

**Authors:** Cong Wang, Xinyu Qiao, Zengli Gao, Lianzhou Jiang, Zhishen Mu

**Affiliations:** ^1^Center of Experimental Instrument, School of Food Science and Technology, Dalian Polytechnic University, Dalian, China; ^2^Inner Mongolia Mengniu Dairy Industry (Group) Co., Ltd., Hohhot, China; ^3^College of Food Science, Northeast Agricultural University, Harbin, China

**Keywords:** dairy industry, components, preparation, functionality, prosperity

## Abstract

Dairy products have become more common in people's daily diets in recent years, and numerous useful components derived from milk are widely employed in the food industry. Milk fat globule membrane (MFGM) is a kind of film that encases milk fat globules, and has been shown to have a high nutritional value. In this work, the protein, lipid, carbohydrate, and other components of MFGM are discussed, and also common separation, preparation, and analysis technologies, physicochemical properties, and functional features of MFGM are reviewed, to provide some guidance for the development and utilization of MFGM.

## Highlights

- The identification of primary components of milk fat globule membrane (MFGM) was compared.
- The separation and preparation technologies of components in MFGM were illustrated.
- The bioactivities and applications of MFGM in functional food industry were reviewed.

## Introduction

Milk fat in milk is secreted by breast cells and exists in the form of fat globules with a diameter of 0.2–15 μM. The milk fat globule membrane (MFGM) is a thin layer that surrounds the fat globules. The content of MFGM in milk was different, and the reports about MFGM accounted for 2–6% of fat globules (1, 2). These contents were reported due to the difference in collection season, collection stage, preparation method, and determination method of raw milk.

The structure of MFGM is constructed with three layers, and the thickness of MFGM is 10–50 nm (3). The inner membrane is a monolayer composed of proteins and polar lipids from the endoplasmic reticulum. It is the membrane that is covered by triglycerides when they are accumulated in the endoplasmic reticulum of breast epithelial cells and released into the cytoplasm in the way of budding and growth; the outer membrane is a double-layer membrane, which is composed of proteins and polar lipids from the plasma membrane at the top of the epithelial cells of the mammary gland (4), which is the fat globule in the cytoplasm. When released by mammary epithelial cells in the manner of budding expansion after reaching a specific size, this membrane is covered. Furthermore, some cytoplasm may persist between the inner and outer membranes after lipoglobulin production.

The research heat was low from MFGM's initial report in 1936 until 1970. MFGM research has steadily increased since the 1970's. The current focus of MFGM research is on the following aspects: composition identification, separation and preparation methods, physical and chemical properties, and functional feature analysis. In recent years, the application of MFGM in the food industry has attracted more and more attention. In this article, the separation, preparation, composition, physicochemical properties, functional properties, and development prospects of MFGM are reviewed.

## Isolation and Preparation

Milk fat globule membrane can be separated and prepared from fresh milk, cream, casein, and butter whey. The yield of MFGM in the separation process is influenced by three primary elements: biological factors, such as raw milk type; physical factors, such as temperature; and chemical factors, such as salt ions. The findings suggest that the heat treatment temperature has a significant effect on the integrity and stability of MFGM in milk. The denaturation of protein in MFGM and its binding with whey protein is induced by heating the milk above 60°C (5). The freezing and thawing will lead to the instability of fat globules, which will cause serious damage to MFGM. The stability of MFGM is also affected by fast air intake during separation. The composition, physical, and chemical characteristics of MFGM isolation are also affected by the separation technique, the kind of raw materials, and the preparation of raw materials. Therefore, to avoid the effects of the above elements on MFGM, it is required to research and improve the separation procedure.

**Figure 1:**
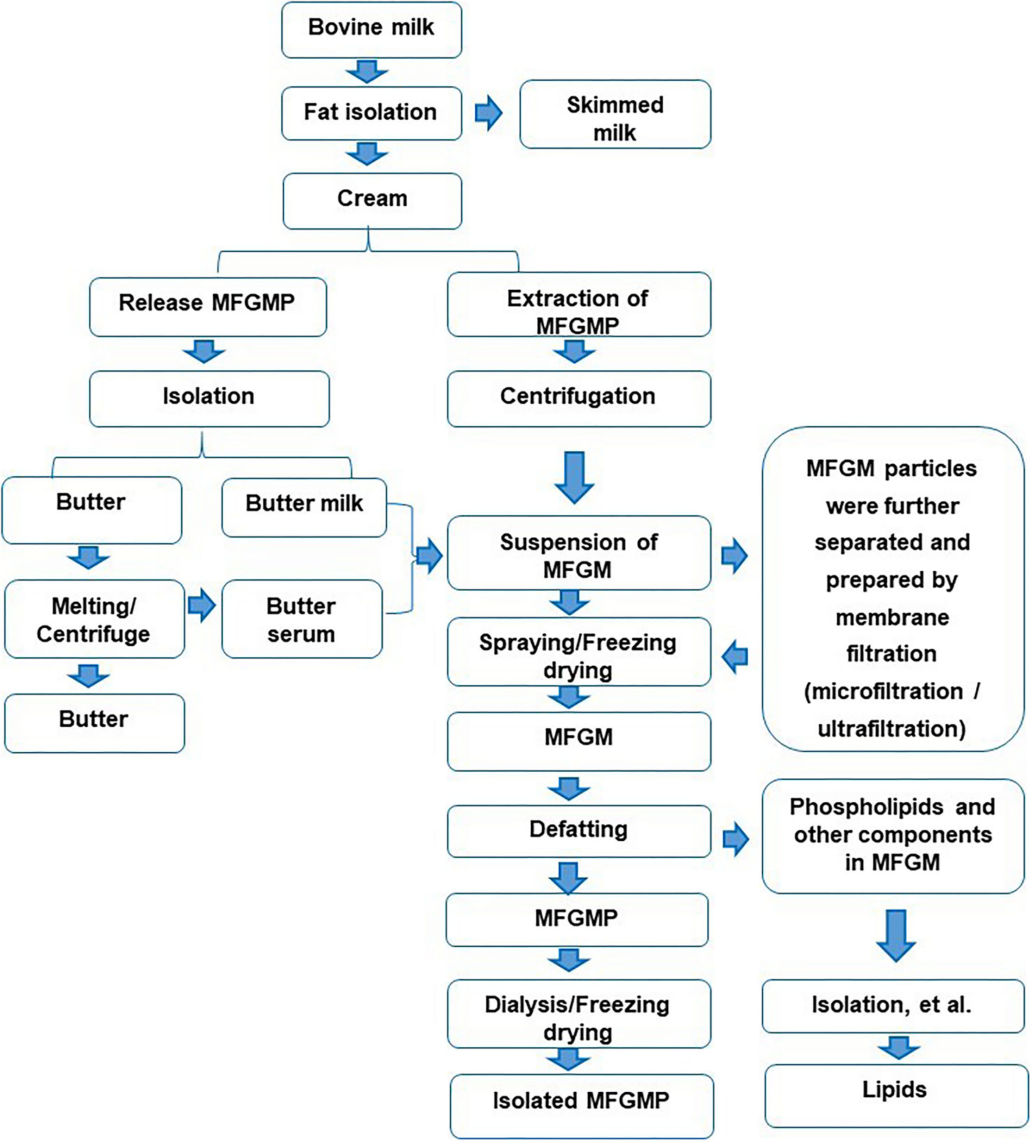
Isolation and separation of the components in Milk fat globule membrane (MFGM) from bovine milk.

There are many reports on the preparation of MFGM with fresh milk as raw material, mainly including degreasing, cream cleaning, MFGM release, collection, drying, and other procedures (6). First, the milk is separated into cream and skimmed milk (7). The cream is washed with water or salt solution of 3–5 times volume for 2–3 times (3), to remove casein and whey protein in cream (8). The washed cream is cooled to 10°C and centrifuged to promote the separation of the fat phase and the water phase (9). The buttermilk and butter particles are obtained by filtering with a filter cloth. After adding water and melting at 40–60°C, the upper layer of centrifugation is butter, and the lower layer is butter whey. The mixture of butter whey and casein is used to obtain MFGM suspension. MFGM suspension is acidified, salted out, or ultracentrifuged to remove the residual lipids, and then dried to obtain MFGM (10). There are also some studies on the preparation of MFGM with casein powder as raw material, which is mainly divided into the following steps: dissolution, washing, centrifugal separation, and drying of casein powder (11). As a whole, the main process of separating MFGM from milk was shown in Figure 1.

In the preparation technologies of MFGM, in addition to physical separation methods, such as salt solution, a filtration device can also be used to remove whey protein and casein in milk (12). In the subsequent infiltration process, reverse osmosis water was added to keep the feed rate unchanged. The MFGM was separated from the reflux by acidification (13).

In the dairy industry, butter whey is usually selected as the main source of MFGM (4). In this process, whey protein and casein are mainly removed from raw materials (14). Since MFGM is similar to casein in size, casein must be removed before microfiltration. In industry, MFGM is usually prepared by a two-step method: first, rennet or casein is used, and then MFGM is obtained by microfiltration. It can be obtained by adding sodium citrate into the cheese milk to remove the casein by microfiltration and then centrifuging at high speed.

## Composition of MFGM

**Figure 2:**
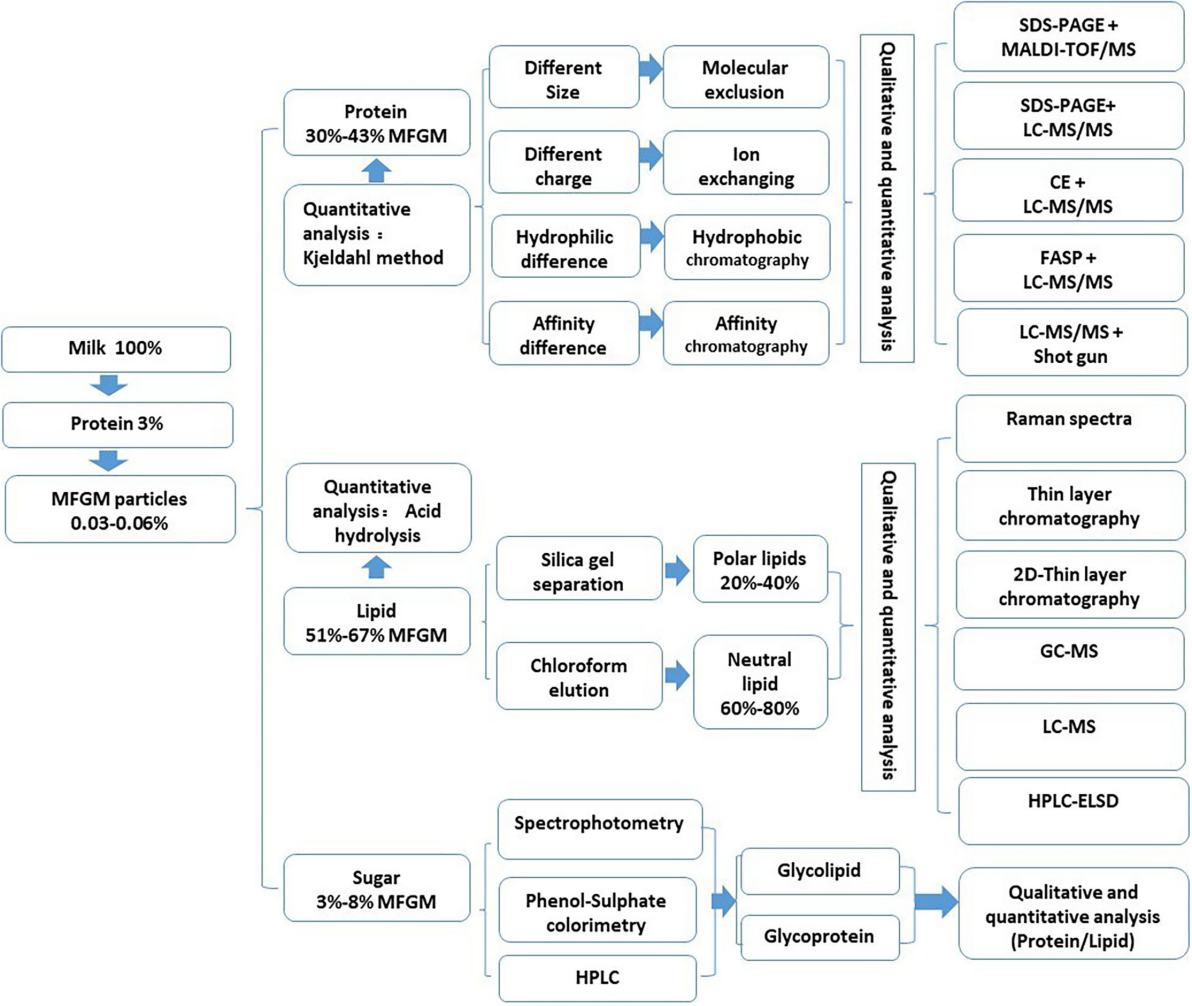
Identification and the quantitative analysis of the components in MFGM.

The composition of MFGM is complex, mainly composed of polar lipids and membrane-specific proteins. Due to different separation, purification, and analysis techniques, the composition of MFGM in the relevant literature is also quite different (15). At the same time, factors, such as lactation stage, season, feed, and fat globule size, also affect the content and composition of MFGM (16). There are many reports on the molecular types of protein components, lipid components, and polysaccharide components in MFGM, but there are great differences among them. The qualitative and quantitative analysis methods of these components are summarized in Figure 2.

### Proteins in MFGM

According to the current publications, the protein content of milk MFGM is about 25–30%. Due to the different separation and preparation conditions and analysis methods of MFGM, the highest protein content in the industrial MFGM powder is about 60%. At the same time, the number of MFGM proteins reported in different studies was also different. Lu et al., identified 169 kinds of MFGM proteins in milk by the filter-aided sample preparation method (FASP) and Nano LC-MS/MS. In addition, 312, 554, 175, and 143 MFGM proteins were identified from human milk, cow milk, goat milk, and yak milk (17). Le et al., used polyacrylamide gel electrophoresis (SDS-PAGE) and liquid chromatography-tandem mass spectrometry (LC-MS/MS) to identify 225 proteins from milk-derived MFGM (2). Michael et al., identified 244 and 133 kinds of proteins in whey protein concentrate and casein concentrate by LC-MS/MS combined with the shotgun method (18).

Among the MFGM proteins, there are 8 kinds with the highest abundance, including MUC1 and MUC1 six glycoproteins, xanthine oxidoreductase (XDH/XO), mucin 15 (MUC15/PAS III), fatty acid transporter (CD36/PAS IV), lactophilic lipoprotein and lectin (pas6/7), and two non-glycosylated proteins: adipogenic differentiation-associated protein (ADPH) and fatty acid-binding protein (FABP).

### Lipids in MFGM

The main lipids in MFGM are polar lipids and neutral lipids (15). The main polar lipids can be divided into five types: phosphatidylcholine (PC: 19.2–37.3%), phosphatidylethanolamine (PE: 19.8–42%), sphingomyelin (SM: 18–34.1%), phosphatidylinositol (PI: 0.6–13.6%), and phosphatidylserine (PS: 1.9–16%) (19). Phosphatidylcholine and sphingomyelin are mainly located in the outer layer of the cell membrane. Phosphatidylethanolamine is mainly located in the outer layer of the cell membrane, and phosphatidylinositol and phosphatidylserine are concentrated in the phospholipid bilayer. Neutral lipids include triglyceride, diglyceride, monoglyceride, cholesterol, and other esters, of which triglyceride is the main part (accounting for 62% of the total lipid) (10). The content of neutral grease in MFGM is variable (56–80%), which largely depends on the separation method used in MFGM separation (20).

### Sugars in MFGM

The carbohydrate in MFGM is mainly combined with proteins or lipids and exists in the form of glycoprotein or glycolipid. Glycolipids can be divided into neutral glycolipids and acid glycolipids (gangliosides). Ceramide is sphingosine, which is linked to fatty acids by an amide bond. Neutral glycolipids are composed of one or more carbohydrate residues connected to ceramide in the lipid portion, including galactosylceramide, glucoceramide, and lacer. Acid glycolipids are composed of oligosaccharides linked by ceramide to at least one sialic acid and various other residues through glycosidic bonds. Glycoproteins include mucin, butyrophilin (BTN), fatty acid transporter (CD36), milk lectin PAS 6 (52 kDa), and PAS 7 (47 kDa) (21).

There are few reports about the content of polysaccharides in MFGM and the differences are large. He et al., determined the mass fraction of polysaccharides in yak MFGM by phenol sulfuric acid method, which was about 0.1 mg/g. Ji et al., determined the mass fraction of polysaccharides from five kinds of mammals, including cow milk (8.54 mg/g), camel milk (5.90 mg/g), buffalo milk (5.06 mg/g), yak MFGM (3.16 mg/g), and goat milk (2.96 mg/g). It can be seen that the mass fraction of polysaccharides in MFGM is related to many factors, such as mammalian species, separation, and analysis methods of MFGM.

## Functional Properties Of MFGM

### Emulsifying

Milk fat globule membrane and its components are amphiphilic, and they are considered to be excellent emulsifiers. The results indicated that the emulsion created with MFGM and MFGM protein had lower viscosity and smaller droplet size than the emulsion prepared with MFGM lipid. The results of heat treatment showed that the emulsion prepared by MFGM was stable at the temperature range of 35–85°C, whereas the emulsion made from MFGM protein became unstable at temperatures above 65°C (22).

The emulsifying performance of MFGM is related to its composition, which is closely related to the raw materials and separation methods of MFGM. It was found that the MFGM rich material separated from the reconstituted milk by microfiltration had a better emulsifying performance by comparing the particle size distribution, viscosity, and stability of different emulsions (23, 24). The discovery of emulsifying properties of MFGM is also helpful to the food industry. Due to the poor bioavailability and absorption of β-carotene in the aqueous phase, its application in the food industry has been greatly restricted. MFGM can be used as an emulsifier to form emulsion with β-carotene, which can improve the dispersion and poor stability of β-carotene in water, so as to improve its bioavailability (25).

### Foaming

Due to the emulsifying properties of MFGM and MFGM protein, adding an appropriate amount of MFGM and MFGM protein in whipping cream will cover fat droplets and form an aggregated network structure, which will cover the air mixed in the whipping process and increase the foaming rate. Within a certain time range, there is a positive correlation between the stirring time and the foaming rate.

### Water Holding

Because of its high water-holding capacity, MFGM may prevent moisture loss and migration in the bread core, which reduces the problem of bread aging and hardening. It can also be used as a bread improver, which can not only increase the nutritional value of bread, but also improve the quality of bread.

## Bioactivities Of MFGM

**Figure 3:**
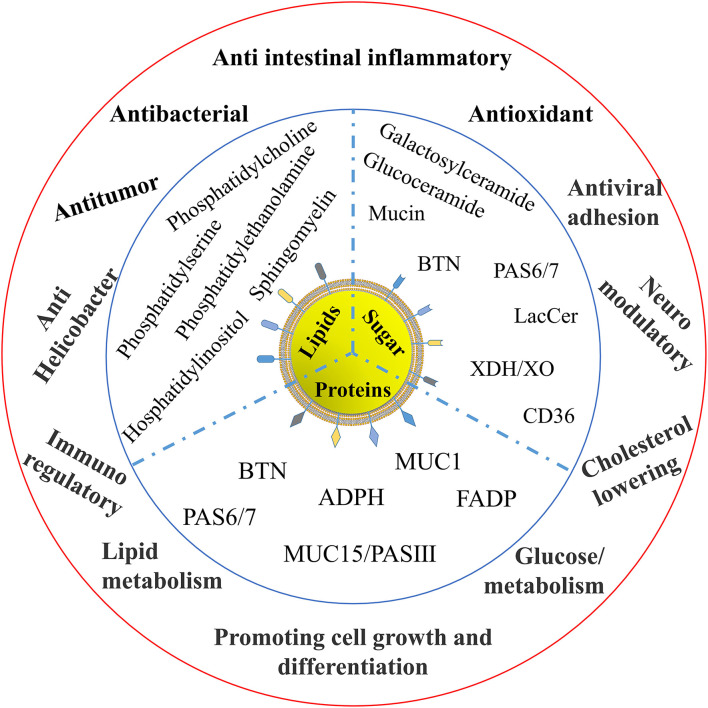
Bioactivities of the components in MFGM.

There are various functional components in MFGM. Reinhardt et al. discovered that 23% of MFGM proteins are involved in membrane or protein transport, 11% in fat transport or metabolism, 7% in protein synthesis, binding, or folding, 9% in carrier protein, 4% in immune protein, and 2% in β- and *x*-casein; the remaining 21% in protein function is unknown (26). These functions are mainly derived from the protein, lipid, and carbohydrate components of the MFGM, as shown in Figure 3. From the current situation reported, the functional characteristics of MFGM are mainly as follows.

### Immunomodulatory Activity

Many components in MFGM have been proved to have many nutritional values. BTN has a positive impact on human central nervous system diseases. BTN has sequence homology with antibodies detected in autistic children's serum. It can participate in the immune regulation of autism, positively regulate autoimmune encephalomyelitis, and affect the pathogenesis of autism. Some studies have also shown that compared with infants who eat skimmed milk powder, MFGM as complementary food can regulate the metabolic abnormalities of malnourished infants, thus improving metabolic regulation and enhancing immunity (27).

### Anti-intestinal Inflammatory Activity

Infants given commercially available formula had a greater incidence of acute otitis media, gastrointestinal, and respiratory tract infections within 1 year of birth, compared with breastfed infants, perhaps because of the absence of MFGM. Studies have shown that supplementing formula milk powder with bovine MFGM can help to reduce the incidence of acute otitis media in infants and the utilization rate of antipyretic drugs and has an immunomodulatory effect on the humoral response of pneumococcal vaccine (28).

In recent years, the research on the protective effect of milk agglutinin on the gastrointestinal tract has become a hot spot. Milk agglutinin can specifically bind with various rotaviruses, inhibit its replication, reduce the apoptosis of intestinal epithelial cells, and prevent rotavirus infection leading to gastroenteritis. Mexican newborn research discovered a link between milk lectin levels in breast milk and infant rotavirus infection.

In addition, glycoprotein, butyric glycoside, lactonectin, and mucus of MFGM have antiviral effects *in vitro*, whereas oligosaccharides can inhibit the binding of a variety of bacteria (including pneumococci) to mucosa (29). Mucin has been proved to reduce the adhesion of Yersinia enterocolitica to the intestinal mucus.

### Antitumor Activity

In the milk of cattle, goat, buffalo, yak, and camel, MFGM of goat and buffalo showed a better effect in inducing apoptosis and reducing the activity of HT-29 cells (30). Colon cancer, breast cancer, and other malignancies are all inhibited by the lipid and protein components of MFGM. Studies have found that the FABP in MFGM can inhibit the growth of breast cancer cells *in vitro* at very low concentrations. Nerve sphingomyelin can induce cancer cells to stop growth, differentiation, and apoptosis through ceramide, sphingosine, and metabolites of colon cancer cells, which can effectively inhibit early and late colon cancer. In human breast cancer cells, the expression of milk agglutinin will be reduced the survival rate of cancer cells. Therefore, MFGM can be used as a nutritional supplement to inhibit the growth of cancer cells (31).

### Promoting Cell Growth and Differentiation

Milk agglutinin can support the growth of epithelial cells. The purified MFGM protein can effectively promote cell growth and inhibit the apoptosis of C_2_C1_2_ cells by upregulating the expression of Akt and mTOR protein kinase. MFGM has the potential to aid in illness prevention and also newborn growth and development (32). Studies have shown that adding MFGM to the diet of infants aged 6–11 months can prevent diarrhea, and adding MFGM to the diet of preschool children can improve the fever of children's language function (33). Feeding SM supplemented milk to preterm newborns with birth weights <1,500 g can compensate for neural development deficits caused by inadequate breast milk, resulting in a favorable influence on the infant's neural development (34). The combination of bovine MFGM and lactoferrin in the diet can not only better approach the bioactive components of breast milk, but also contribute to the cognitive, gastrointestinal, and respiratory health of infants (35).

### Other Activities of MFGM

The MFGM differential proteins of yak and cow were shown to be involved in glycolipid metabolism, immunological regulation, antioxidant activity, anticancer, neuromodulation, antibacteria, and viral adhesion in marker-free proteomics. Both differential protein and protein-related genes predicted that yak and bovine MFGM protein had the biological function of regulating glucose and lipid metabolism. *In vitro* experiments confirmed that yak and bovine MFGM protein could reduce lipid accumulation and increase glucose uptake in HepG2 cells. The MFGM protein of yak and cattle has the higher lipid-lowering ability and increasing glucose uptake ability, which are respectively related to different MFGM proteins related to glucose and lipid metabolism in yak and cattle (36).

The elderly's feeling of balance, strength, and sensitivity can be improved by combining MFGM consumption with adequate exercise (37). Furthermore, the inclusion of MFGM may aid in the treatment of dyskinesia syndrome. Sphingolipids in MFGM can not only treat and improve cardiovascular diseases, but also participate in the inflammatory process of atherosclerosis and insulin resistance. Therefore, it can be inferred that dietary sphingolipids have great potential for the treatment of various aspects of metabolic syndromes, such as dyslipidemia, insulin resistance, and cardiovascular diseases.

## Prospective

With the deep understanding of the composition and properties of MFGM, the nutritional function, growth and development of different populations, disease prevention, and other functional characteristics of MFGM have been gradually discovered, but the nutritional and functional characteristics of specific components of MFGM still need to be studied further. At present, infant formula with MFGM concentrate has been launched in the market. Special medical purpose formula food, special food, and functional food containing MFGM still have a sizable market. Furthermore, the research heat of the MFGM is increasing, and the fine separation and preparation technology is improving.

## Author Contributions

CW, ZG, and LJ contributed to conception and design of the study. XQ organized the figures. ZM performed the statistical analysis. CW wrote the first draft of the manuscript. ZG and XQ wrote sections of the manuscript. All authors contributed to manuscript revision, read, and approved the submitted version.

## Funding

This study was financially supported by the Basic Research Program of Liaoning Education Department (2017J080) and the National Natural Science Foundation of China (31371805).

## Conflict of Interest

CW is a full-time faculty in the Dalian Polytechnic University and she directed a research work funded by the Inner Mongolia Mengniu Dairy Industry (Group) Co., Ltd., in which ZG and ZM engaged. The remaining authors declare that the research was conducted in the absence of any commercial or financial relationships that could be construed as a potential conflict of interest.

## Publisher's Note

All claims expressed in this article are solely those of the authors and do not necessarily represent those of their affiliated organizations, or those of the publisher, the editors and the reviewers. Any product that may be evaluated in this article, or claim that may be made by its manufacturer, is not guaranteed or endorsed by the publisher.
