# Supplementary material for: Advancement on Milk Fat Globule Membrane: Separation, Identification, and Functional Properties
Source: Front Nutr. 2022 Jan 28;8:807284. doi: 10.3389/fnut.2021.807284 (PMC8832003; doi:10.3389/fnut.2021.807284)
Supplement: Supplementary file 1 [file Presentation_1.PPTX]

## Slide 1
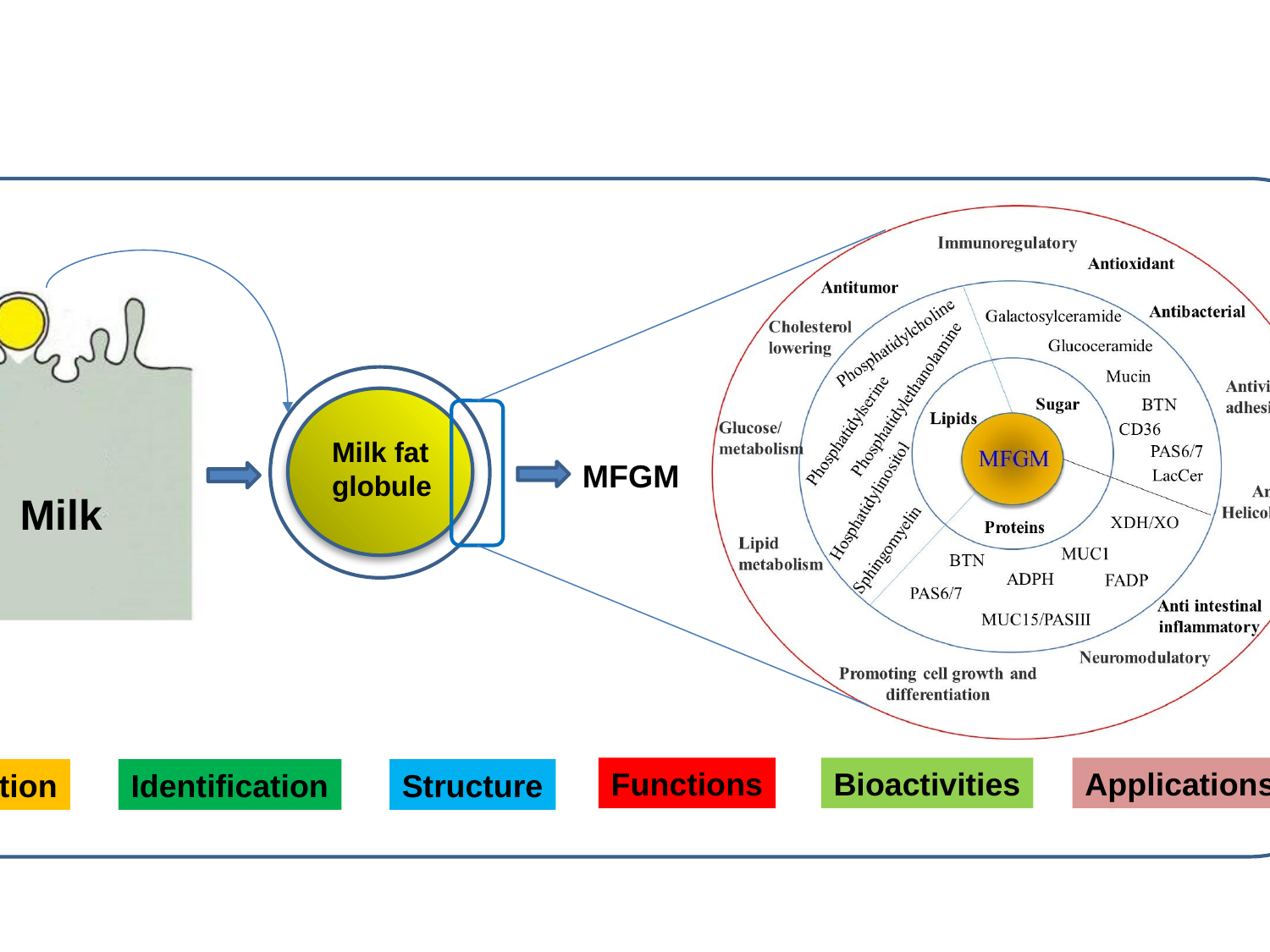

Milk fat
globule
MFGM
Milk
Functions
Bioactivities
Applications
Isolation
Identification
Structure
